# The Abortive Treatment of Gonorrhea in the Male

**Published:** 1904-10

**Authors:** 


### THE ABORTIVE TREATMENT OF GONORRHEA IN THE MALE.*

The attempts to devise an abortive treatment of gonorrhea did not originate in the immediate past, but were already tried in the days preceding the discovery of the gonococcus, by Ricord, Diday, and others. To those familiar with the severity of the complications and sequelæ of gonorrhea, as well as its wide spread through all the strata of society, the possibility of aborting this disease, in its earliest hours, or days, must appear almost as a blessing, if the cure can be effected without harm to the patient. There are two methods of achieving this result now at our disposal, viz., the *prophylactic*, by means of which those gonococci which may have obtained lodgment in the urethra are killed immediately, or shortly after the infective coitus, and the development of an inflammation prevented; secondly, the *abortive*, by means of which the already developed gonorrheal urethritis is cured in a few days, and the pathological changes, which such an inflammation produces are reduced to a minimum.

The prophylaxis, which is being more and more adopted, is the ideal treatment. It is possible in either of two ways: either through the use of a reliable condom (preferably of fish-skin), or through instillations, injections, etc., of the gonococcicide drugs, immediately after coitus. Thus Haussman, Kopp and Blokusewski recommend the instillation of a few drops of a two per cent. solution of nitrate of silver. Probably the greatest credit, in this direction, is due to E. R. W. Frank, and Welander, for their studies in the prevention of gonorrhea, by means of protargol solution. Both working independently of each other, succeeded though their inoculation experiments with fresh gonorrheal pus, in demonstrating conclusively that it is possible to *prevent* gonorrhea. I have adopted the prophylactic measures, in my practice, and rec-

*Read by Frederick Bierhoff, M. D., New York, before the German Medical Society (New York), December 7, 1903; Harlem Medical Association, January 6, 1904.

ommend my patients to instil 2 to 3 drops of a 10 to 20 per cent. solution of protargol in glycerin into the fossa navicularis, as soon as possible after the suspicious coitus. In one case it was possible for me to discover the gonorrheal infection within ten hours after the suspicious intercourse, and to abort the disease with a single injection of 8 c.c. of a two-per-cent. protargol-glycerin-water solution. This case, which I have not included among the thirty cases referred to further on in this article, I was able to control exactly, and I found, on the first examination, *distinct*, although few, gonococci, in the urethral scraping; at several points there were already distinctly gonococcus-bearing pus-cells. Control examinations were made after one, four, eight, twenty-four and forty-eight hours. Already after *one hour* the secretion, which was called forth profusely by the injection, was free of all germs, and remained so, which fact I was able to determine by repeated subsequent control-examinations, during a period of several months. The discharge rapidly lost its purulent character, after the cessation of treatment, to become purely epithelial within twenty-four hours, and disappeared entirely after another twenty-four hours.

As is the case with the prophylactic, so the possibility of an abortive treatment depends upon the fact that the gonococci, in the earliest days, when not abnormally virulent, or brought to virulence through irritants, or injury, or a markedly dimished power of resistance of the tissues, have a tendency to remain confined to the fossa navicularis, because the walls of the fossa navicularis are covered with stratified, squamous epithelium, which, as is well known, is resistant to the action of this germ. I am of the opinion that many a gonorrheal infection of low virulence dies out, upon the epithelium of the fossa navicularis, without its bearer having been aware of the fact of the infection. As a result, in all probability also of this power of resistance, the changes in this part of the urethra, demonstrable after the cure of a gonorrhea, are very slight.

If we see a case of gonorrhea early, that is, at the very beginning of the appearance of the visible discharge, then we notice, in addition to the usual reddening of the urethral orifice, beginning hypersensitiveness and a rapid increase in the number of leucocytes, as proofs of the fact that the infection has passed beyond the fossa navicularis, and has begun to develop on and penetrate between the cylindrical epithelia of the pars cavernosa. We also notice that the gonococci which, in the first hours after infection, and so long as the process had been confined to the fossa navicularis, had almost entirely been *extracellular*, now more and more come to lie *intracellular* (I refer to secretion obtained with the loop, just within the meatus, without the exertion of any pressure or stripping of the urethra, the secretion then being gently spread out, on the slide, with the loop. Not by drawing it off with another slide). The more the process extends over the pars cavernosa, and the deeper into the tissues that the infection penetrates, the less are, in my estimation, the chances for the success of any abortive treatment. The sooner one begins with the treatment, the greater are the chances of success, in the absence of complicating conditions. The complicating conditions are the presence of remains of pre-existing disease; for instance, chronic gonorrheal infection of the lacunæ, glands of the Littré, or prostate; and congenital malformations of the urethra, such as paraurethral passages, or abnormal width, or depth of the lacunæ.

The method of treatment has differed, with different investigators. Ricord, Debenay, Diday and others employed injections of nitrate of silver, in solutions up to five per cent. in strength. Delefosse instils a two to two and one-half per cent. solution of the drug, by means of the Guyon instillation syringe, into the urethra, applying it to the membrane, from the bulb to the meatus. This is repeated on the following day. If, on the third day, gonococci are still present, a two-thirds to one-half per cent. solution is employed. Pontopiddan reports on 160 cases treated with the two per cent. solution. He applied several drops to the fossa navicularis, after the patient had urinated. In one-third of his cases the disease was aborted; in the rest, the disease ran a mild course. Ullman injected 3 to 5 cc. of a two per cent. solution, 4 to 5 cm. into the urethra, the solution being retained for two minutes; he also reports positive results. Welander recommends, in fresh, at most two to three days' old cases, that the urethra be first wiped clean, and, following this, the injection of a two per cent. solution, which is retained for two minutes.

Fèleki recommends, in fresh cases, the penciling of the urethral wall with five per cent. solution, through the endoscope. He states that, where the secretion was slight and of a serous character, one application sufficed; in other cases it had to be repeated, after two to three days. He claims to have had no complications.

Engelbrecht recommends irrigations, according to the Janet method, with 500 to 600 cc. of a one-fifth to one-third per cent. nitrate of silver solution, after preceding cocainization of the urethra. He claims to have cured nine-tenths of his cases in two days, after four applications. Neisser recommends early irrigations with silver nitrate solution of 1:3,000 to 1:1,000 strength; Finger and Jadassohn report that they have had no success with this method. Tarnowsky is reported to have cured 40 to 50 per cent. of his cases of acute gonorrhea, in four days, by means of nitrate of silver solution; but reports that, in those cases which resulted negatively, the symptoms of inflammation were increased to such a degree that the treatment had to be abandoned. According to the reports of most of the observers, the reaction following the abortive attempts with nitrate of silver solution is very severe and, at times, severe complications arise, resulting even in death, as in a case reported by Cullerier and Guyon.

Another variety of the abortive treatment is the application of Janet's method and its modifications. The irrigations with permanganate of pottassium are recommended by Janet himself, by Valentine, Goldberg and others; Wossidlo, Horwitz, Weiss and Christian express themselves against the method, as an abortive.

Since we have come into possession of the various drugs, such as protargol, albargin, ichthargan, argyrol, etc., which destroy the gonococcus where they can reach it, while they irritate the mucous membrane but little, various investigators have tried the abortive treatment with these remedies. Thus Ahlstrom reports on 100 cases which he treated abortively. Twice daily, for the first four or five days, he injected 5 to 10 cc. of a two to four per cent. solution of protargol. For the next three to five days the patients themselves injected with a one to two per cent. solution, which they retained in the urethra for ten to fifteen minutes.

In only 13 per cent. was there a negative result; in 8 per cent he saw complications. He must have had model patients to deal with, since the large majority of patients complain, even with injections of a one per cent. solution, of almost unbearable pain, when the solutions are to be retained in the urethra for ten minutes. Or are our American patients, perhaps, weaklings, when compared to the European? I have found, too, that with such strong solutions the other symptoms of irritation are so severe that I should hardly recommend them. Neisser and Blaschko speak in favor of the early treatment of gonorrhea, with three to four per cent. protargol solution; Wossidlo, on the contrary, saw no benefit from it, but rather, unpleasant results.

Casper states that he can not say of a single case that the begining gonorrhea was checked by means of the abortive treatment, and would, therefore, recommend its abandonment under all circumstances. Fon Zeissl states that he can not recommend it. Finger states that he believes the rapid cure of gonorrhea to be an impossibility.

Thus we see that the views for and against the abortive treatment of gonorrhea are varied.

Before the urological division of the Thirteenth International Medical Congress, Frank reported upon a series of 60 cases which he had treated according to the abortive method devised by Lewin and himself. The method, whose application and trial I had the pleasure of witnessing, during their experiments, is reported as follows: "After the microscopical diagnosis has been made, the patient passes water. If the second portion of the urine is clear, the anterior urethra is next irrigated under light pressure, with one-quarter per cent. protargol solution, until the fluid returns clear. If the patient is very sensitive, a solution of cocaine, to which, as a precaution, some protargol has been added, is injected into the urethra. Following this we make a large irrigation of both portions of the urethra, with a similar protargol solution, according to Janet's method. The solution is allowed to flow until the patient notices a desire to urinate; that is about one-quarter to one-half liter, whereupon he empties the bladder. This procedure is repeated upon the following two days." Frank reports upon

60 cases treated by this method. In 27 (45 per cent.) the result was positive; that is, after three irrigations (in one instance after a single one) the gonococci were definitely destroyed. In 26 cases the result was negative. In three of these negative cases gonorrheal infection of paraurethral passages was discovered; after incision of the passages, further two to three irrigations cured the cases. In two other cases, the presence of congenital fold and valves, with narrow meatus, was the cause of the negative result. In 21 cases the prostate was infected; and Frank believed that this infection had already existed at the time the attempts to abort the disease were begun. The remaining seven cases withdrew from treatment, to present themselves later as cured.

My observations, made at the time of Frank's experiments, determined me to test this method of abortive treatment. Although I have, as a result of circumstances, slightly modified the plan of treatment to meet the requirements of my patients, it remains, in principle, that laid down by Frank and Lewin. In order to be able to control the cases more exactly, I employed it only in the cases of intelligent private patients, who, as soon as they realized the aim of the measures, were willing to help me with all their power. The method employed was as follows: A microscopic examination of the secretion was first made. If the discharge was slight, and if the majority of the gonococci still were extra-cellular, then the protargol solution was employed in the strength of one-sixth to one-third per cent. If the discharge was at all pronounced, or if the greater part of the gonococci were intra-cellular, then a one-third to one-half per cent. solution was used. The method was employed, naturally, only in those cases in which the second urine was clear. After urination, the urethra was anesthetized by an injection of a mixture of 4 cc. of a one per cent. cocaine solution and 4 cc. of a one per cent. protargol solution. After this, the anterior urethra was cleansed with 150 cc. of the protargol solution. Following this, an irrigation of the whole urethra was made according to Janet's method, with 150 cc. of the solution. The patient then emptied his bladder of the irrigated fluid. This irrigation of the entire urethra, with immediate emptying of the fluid, was repeated from one to three times in the same sitting, so that the urethra was flushed four to eight times. In addition, the patient was given a solution of protargol, one-half per cent., with which he was instructed to inject three to five times during the day, and to retain the fluid ten minutes each time. During the succeeding days, if the gonococci had disappeared, the strength of the solution and the quantity of fluid injected were diminished, to be suspended, if the result was positive, on the fourth or fifth day, at the latest. The injections by the patient were also diminished and suspended in a similar manner. Then followed the usual provocative tests and control examinations. I will state, at this point, that I consider the continued absence of discharge and the absence of gonococci from the shreds (which should be only minimal and chiefly epithelial), from the prostatic secretion (which must also be free of pus-cells), and from the urinary sediment, and this in spite of the tests of cessation of treatment, indulgence in alcoholics, irritating injections, and coitus condomatus, to be a proof of cure if, after control examinations covering a later period of several weeks, the patient still remains free of discharge and gonococci. Cultures, I believe, should be employed in every doubtful case. I do not, however, believe them to be an absolute necessity to determine the fact of a cure.

In all, I have employed the method in thirty cases (up to November 1, 1903). In fifteen cases (50 per cent.) the result was positive; in fifteen (also 50 per cent.) negative.

[Here follow detailed statements as to the day of the disappearance of the gonococci, etc.] The author concludes:

I know that the number of my cases is too small to allow me to draw definite conclusions; neither do I wish to claim that the method mentioned in this article is the best one. I only wish to present the experiences I have gained through its application as proof of the facts: That the abortive treatment of gonorrhea is possible; that if it be employed *early*, the percentage of cases in which a positive result may be obtained is a fairly large one; that when one employs only those drugs which, while they kill the gonococci, do not injure the mucous membrane, the percentage of complications will, most likely, be smaller than has heretofore been supposed; and, finally, that, should the attempt to abort the disease fail, the patient will not have been injured.

